# Supplementary material for: Improving the robustness of the Sequentially Optimized Reconstruction Strategy (SORS) for visual field testing
Source: PLoS One. 2024 Apr 4;19(4):e0301419. doi: 10.1371/journal.pone.0301419 (PMC10994286; doi:10.1371/journal.pone.0301419)
Supplement: S1 Fig — (PDF) [file pone.0301419.s001.pdf]

Supporting Information

S1. Results using staircase instead of ZEST: overall performance

Here we performed the simulation as in the main text but swapped the ZEST single-location thresholding algorithm with 4-2 double staircase algorithm. The results are similar and demonstrate robustness against small training size in the dimensionality reduction technique (TTPCR).

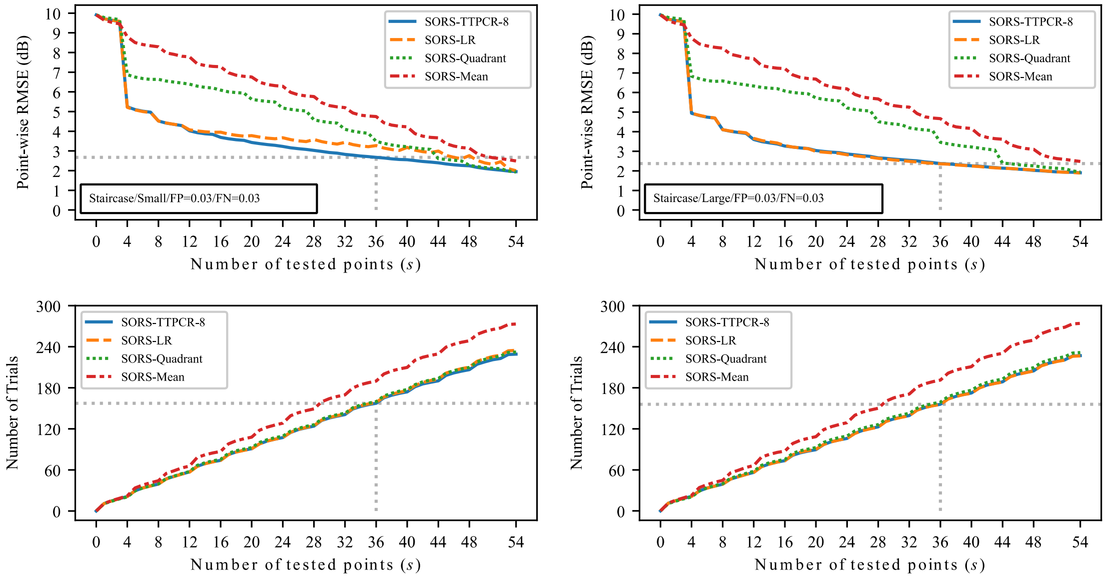

Figure S1 Cross-validation performance using 4-2 staircase in a subject with FP=3% and FN=3%
