# Supplementary material for: Improving the robustness of the Sequentially Optimized Reconstruction Strategy (SORS) for visual field testing
Source: PLoS One. 2024 Apr 4;19(4):e0301419. doi: 10.1371/journal.pone.0301419 (PMC10994286; doi:10.1371/journal.pone.0301419)
Supplement: S2 Fig — (PDF) [file pone.0301419.s002.pdf]

S2. Results using staircase instead of ZEST: distribution of RMSE and test durations

Continuing from the staircase example above, Figure S2 illustrates the distribution of RMSE and test durations.

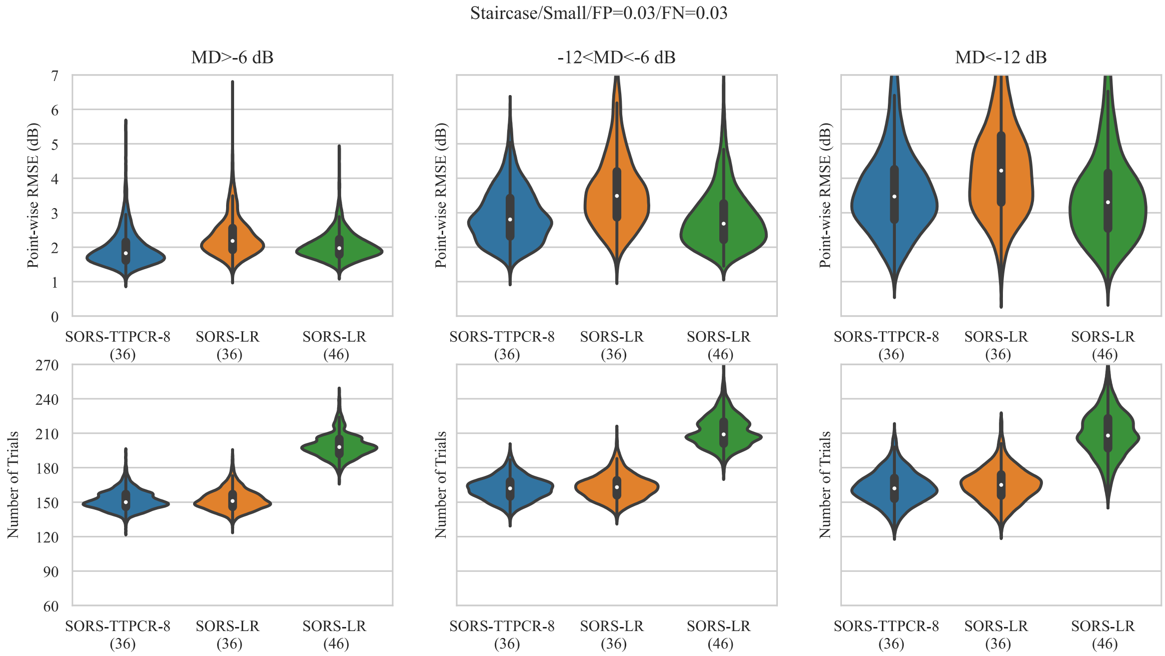

Figure S2 Distribution of point-wise RMSE and test duration after training on a small dataset and using in different disease severities.
