# Supplementary material for: Improving the robustness of the Sequentially Optimized Reconstruction Strategy (SORS) for visual field testing
Source: PLoS One. 2024 Apr 4;19(4):e0301419. doi: 10.1371/journal.pone.0301419 (PMC10994286; doi:10.1371/journal.pone.0301419)
Supplement: S3 Fig — (PDF) [file pone.0301419.s003.pdf]

S3. Results in higher false positive and false negative rates (FP=15%, FN=3%)

When simulated with a high false positive responder (FP=15%, FN=3%, Figure S3), our observations for the robustness of the TTPCR method remains the same.

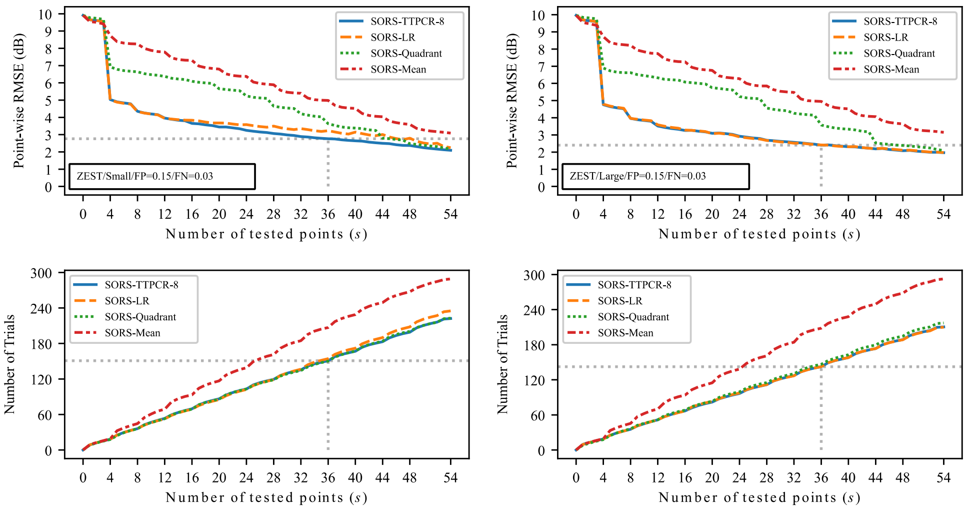

Figure S3 Cross-validation performance using ZEST in a subject with FP=15% and FN=3%
