# Supplementary material for: Improving the robustness of the Sequentially Optimized Reconstruction Strategy (SORS) for visual field testing
Source: PLoS One. 2024 Apr 4;19(4):e0301419. doi: 10.1371/journal.pone.0301419 (PMC10994286; doi:10.1371/journal.pone.0301419)
Supplement: S4 Fig — (PDF) [file pone.0301419.s004.pdf]

S4. Results in higher false positive and false negative rates (FP=15%, FN=15%)

Similar to the example above, results from a high false positive and negative responder (FP=15%, FN=15%) is shown in Figure S4, with similar observations of the robustness of the TTPCR method.

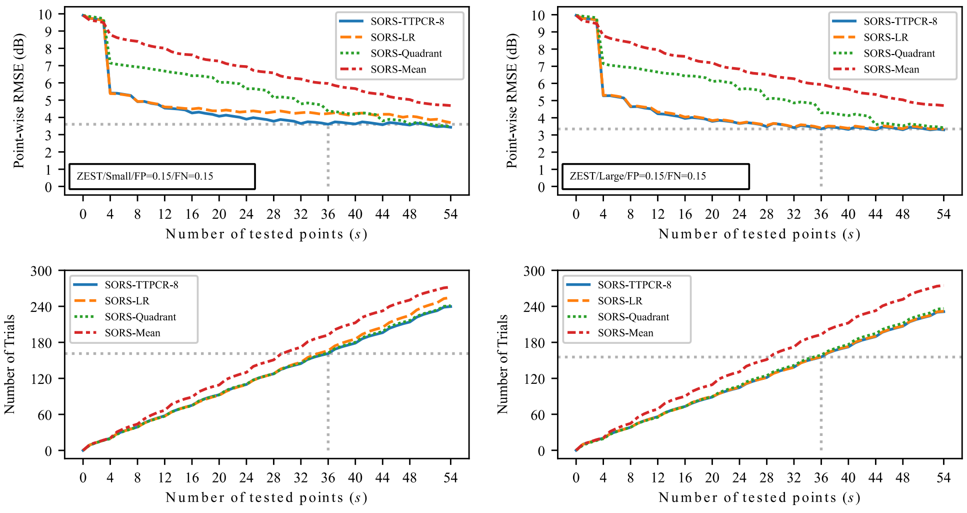

Figure S4 Cross-validation performance using ZEST in a subject with FP=15% and FN=15%
