# Supplementary material for: Improving the robustness of the Sequentially Optimized Reconstruction Strategy (SORS) for visual field testing
Source: PLoS One. 2024 Apr 4;19(4):e0301419. doi: 10.1371/journal.pone.0301419 (PMC10994286; doi:10.1371/journal.pone.0301419)
Supplement: S5 Fig — The autoencoder was trained on the full dataset and tested on all, mild (MD>−6 dB), moderate (−12<MD<−6 dB), and severe (MD<−12 dB) data. The autoencoder has the following architecture: input→54 hidden units→n-dimensional embedding→54 hidden units→reconstructed input. (PDF) [file pone.0301419.s005.pdf]

## *S5. Reconstruction performance of autoencoder*

The reconstruction performance plot of Figure 5 is recreated here but for a 4-layer non-linear autoencoder architecture. The performance is similar to the linear principal component analysis method and worse when a large number of units are used in the embedding layer. This suggests that linear models are efficient models for the visual field reconstruction task.

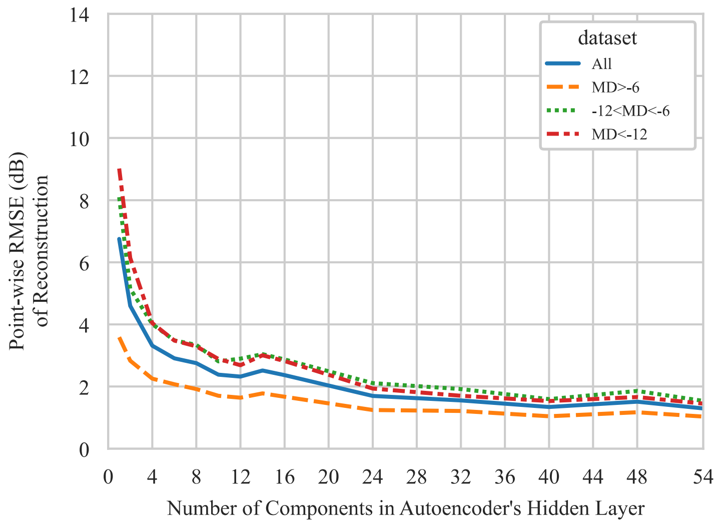


*Figure S5 Precision of autoencoder reconstruction on the Rotterdam dataset as a function of number of principal components used. The autoencoder was trained on the full dataset and tested on all, mild (MD>−6 dB), moderate (−12<MD<−6 dB), and severe (MD<−12 dB) data. The autoencoder has the following architecture: input→54 hidden units→n-dimensional embedding→54 hidden units→reconstructed input.*
